# Supplementary figures and images for: On the treatment effect heterogeneity of antidepressants in major depression: A Bayesian meta-analysis and simulation study
Source: PLoS One. 2020 Nov 11;15(11):e0241497. doi: 10.1371/journal.pone.0241497 (PMC7657525; doi:10.1371/journal.pone.0241497)

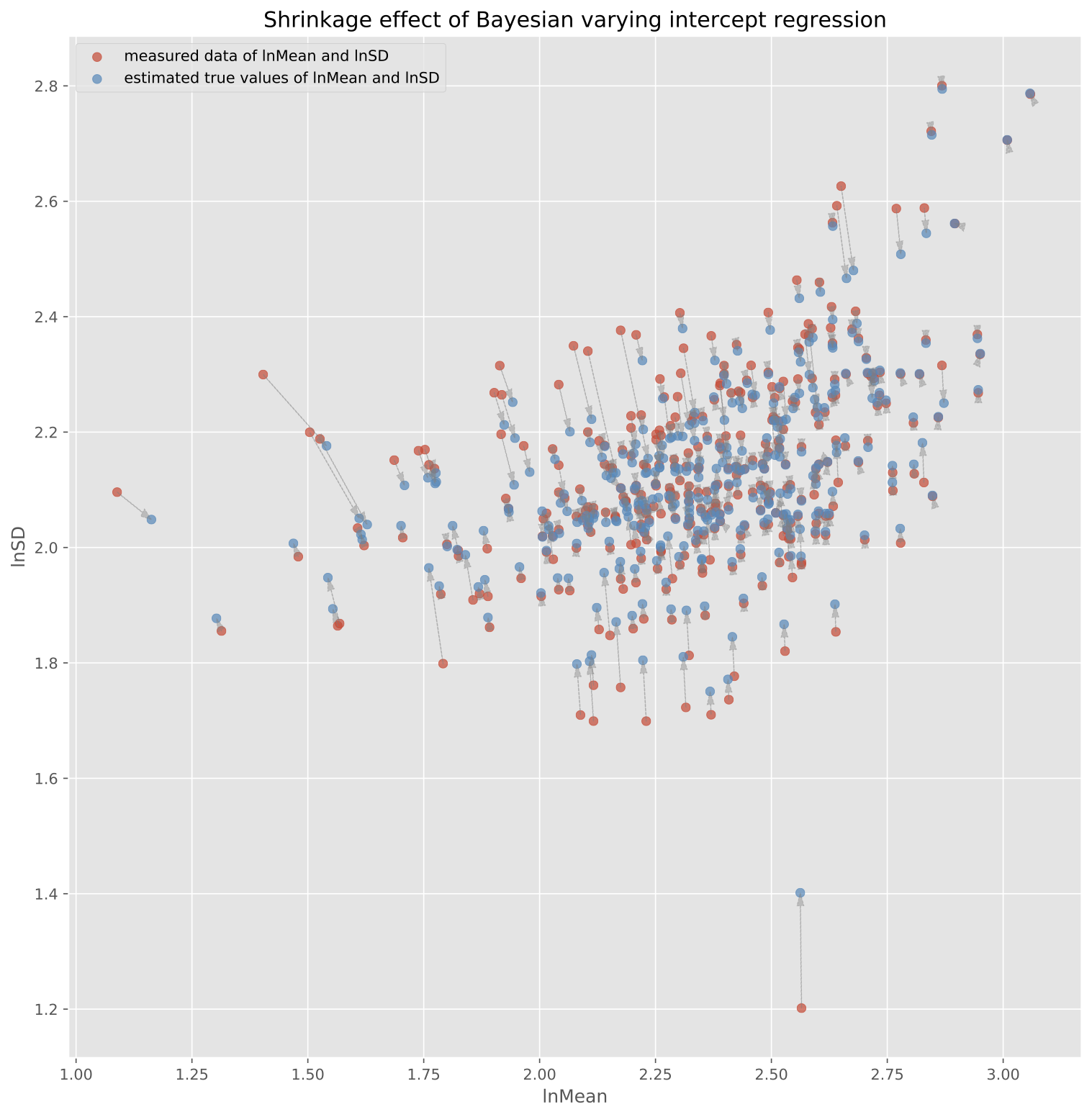

Supplement: S2 Fig — (TIFF) [file pone.0241497.s004.tiff]

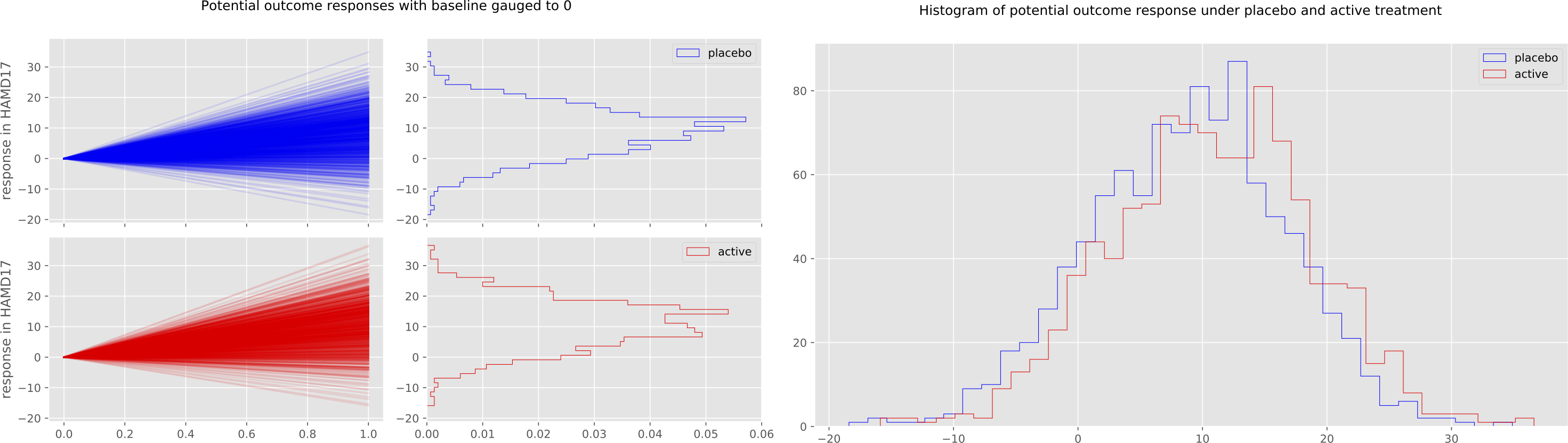

Supplement: S3 Fig — (TIFF) [file pone.0241497.s005.tiff]

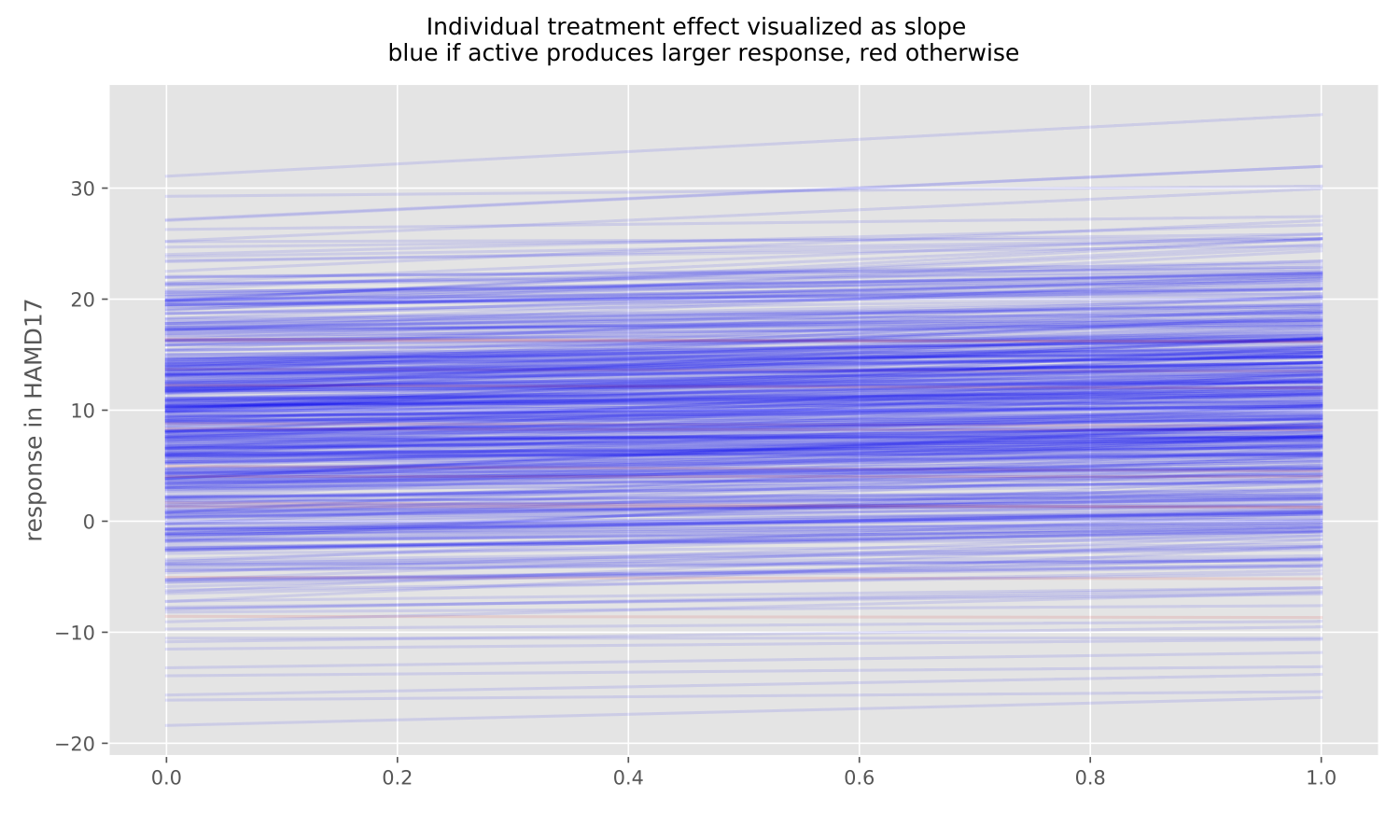

Supplement: S4 Fig — (TIFF) [file pone.0241497.s006.tiff]

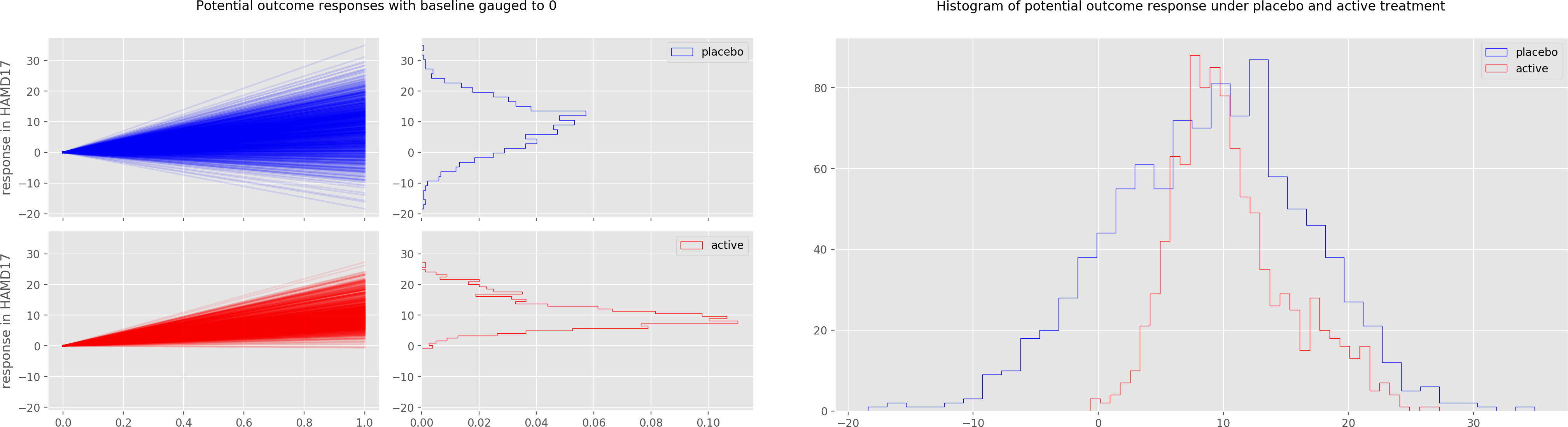

Supplement: S5 Fig — (TIFF) [file pone.0241497.s007.tiff]

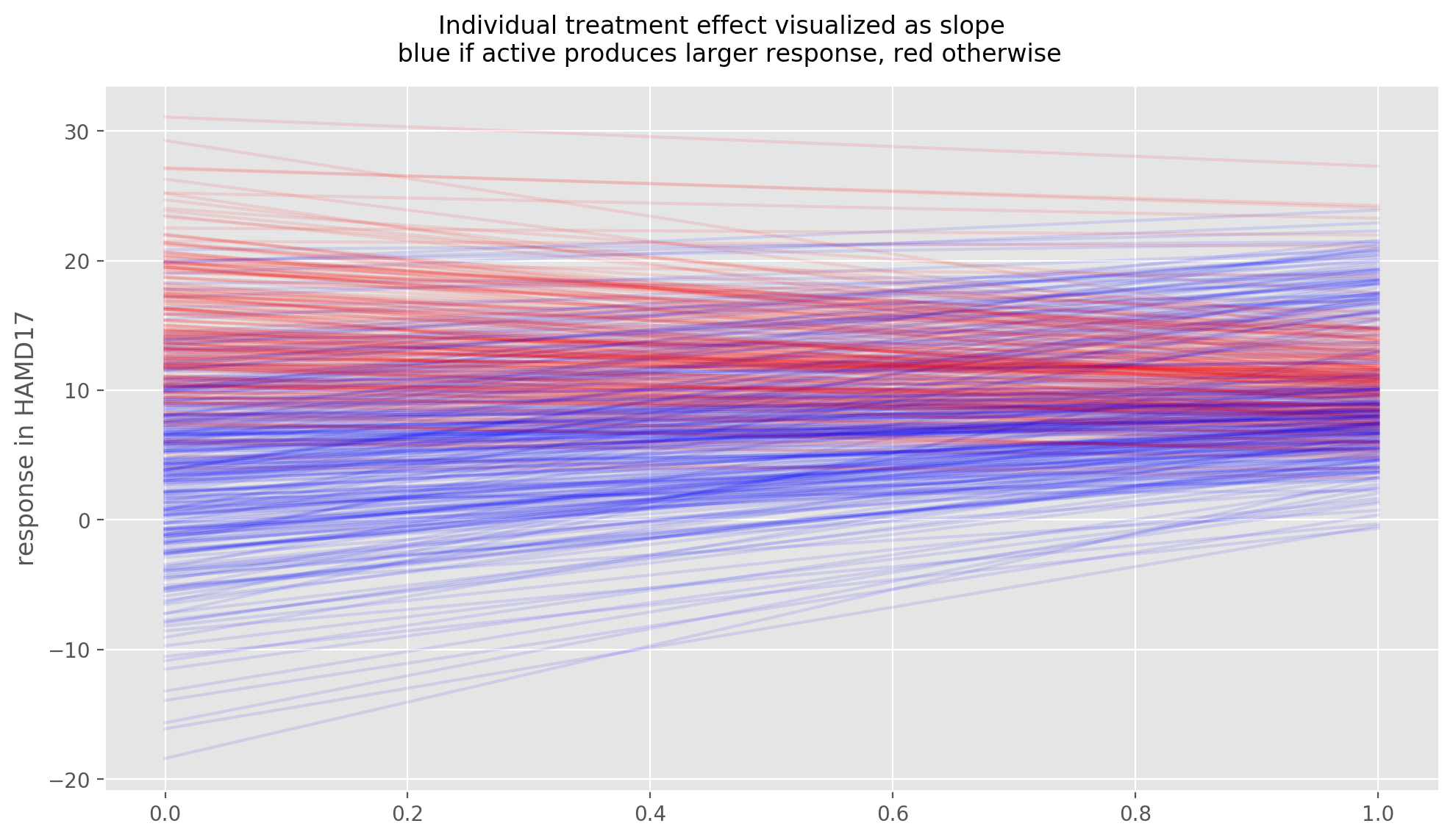

Supplement: S6 Fig — (TIFF) [file pone.0241497.s008.tiff]
