# Supplementary material for: On the treatment effect heterogeneity of antidepressants in major depression: A Bayesian meta-analysis and simulation study
Source: PLoS One. 2020 Nov 11;15(11):e0241497. doi: 10.1371/journal.pone.0241497 (PMC7657525; doi:10.1371/journal.pone.0241497)
Supplement: S1 File — (DOCX) [file pone.0241497.s010.docx]

**On the treatment effect heterogeneity of antidepressants in major depression.**

**A Bayesian meta-analysis**

*Insert supplementary figure 1 here*

**S1 Fig:** **Frequency of depression scales** **used in included studies.**

Boxplot depicting the number of included studies per respective depression scale.

|  | **Max value** | **Reference** |
| --- | --- | --- |
| **HAMD17** | 52 | <http://www.ids-qids.org/interpretation.html>  https://academic.oup.com/occmed/article/65/4/340/1377801 |
| **HAMD21** | 64 | <http://www.ids-qids.org/interpretation.html> |
| **HAMD24** | 75 | <http://www.ids-qids.org/interpretation.html> |
| **HAMD29** | 87 | Imputed from HAMD24 and HAMD31 |
| **HAMD31** | 93 | https://link.springer.com/content/pdf/10.1007/978-1-59745-387-5.pdf |
| **MADRS** | 60 | https://www.ncbi.nlm.nih.gov/pmc/articles/PMC2151980/ |
| **HAMDunspecified** | n/a |  |
| **IDSIVR30** | 84 | <http://www.ids-qids.org/interpretation.html> |

**S1 Table: Max values for respective depression scales.**

|  | **Coefficient** | **SE** | **t** | **P>\|t\|** | **95% CI** |
| --- | --- | --- | --- | --- | --- |
| **intercept** | 1.55 | 0.07 | 21.93 | 0.00 | [1.41, 1.69] |
| **slope** | 0.25 | 0.03 | 8.19 | 0.00 | [0.19, 0.30] |

**S2 Table: Slope coefficient for simple linear association**.

Coefficients for simple linear regression of between lnMean and lnSD of response, not taking into account the existence of different depression scales. The slope coefficient equals to roughly 0.25. SE: Standard error. CI: confidence interval. t: t-value. P: probability.

*Insert supplementary figure 2 here*

**S2 Fig: Shrinkage effect of Bayesian multi-level meta-regression model.**

The red dots represent the data points and the blue dots the posterior estimates of their true values as estimated by the random-effects meta-regression (REMR) model.

|  | **Mean** | **Median** | **2.5% HPD** | **97.5% HPD** |
| --- | --- | --- | --- | --- |
| **e^μ^** | 1.0 | 1.0 | 0.98 | 1.02 |
| **𝛽** | 0.04 | 0.04 | -0.03 | 0.12 |

**S3 Table:** **Posterior statistics for the e^μ^ and 𝛽 coefficients of the REMR.**

Mean, median and 95% HPD interval for the e^μ^ and the 𝛽 coefficient of the meta-regression model, where: $VR\approx e^{\mu}*{RR}^{\beta}$ ($lnVR\approx\mu+ \beta*\ln RR$). e^μ^ corresponds to the direct effect, RR^𝛽^ to the indirect effect. VR: Variability ratio, RR: Response ratio.

|  | **WAIC** | **pWAIC** | **dWAIC** | **Weight** | **SE** | **dSE** | **Warning** | **WAIC scale** |
| --- | --- | --- | --- | --- | --- | --- | --- | --- |
| **REMR lnVR** | 179.94 | 3.82 | 0 | 0 | 36.81 | 0 | 1 | Log |
| **REMA lnVR** | 178.49 | 3.07 | 1.45 | 0 | 38.13 | 1.43 | 1 | Log |
| **FEMA lnVR** | 176.84 | 0.64 | 3.10 | 0.99 | 40.61 | 3.86 | 0 | Log |
| **REMA lnCVR** | 176.84 | 53.08 | 137.11 | 0.01 | 29.26 | 11.79 | 1 | Log |
| **FEMA lnCVR** | 39.24 | 1.47 | 140.70 | 0.00 | 31.46 | 13.14 | 0 | Log |

**S4 Table:** **WAIC statistics for the different models.**

The widely applicable information criterion (WAIC) score signifies the pointwise prediction accuracy of fitted Bayesian models. Here, higher values of WAIC indicate a better out-of-sample predictive fit (“better” model). pWAIC: Estimated effective number of parameters. dWAIC: Relative difference between each WAIC and the lowest WAIC. Best model has value 0. Weight: Corresponds to the relative probability of each model. SE: Standard error of the WAIC estimate. dSE: Standard error of the difference in WAIC between each model and the top-ranked model. Warning: A value of 1 indicates that the computation of the WAIC may not be reliable. REMR: Random-effects meta-regression, REMA: Random-effects meta-analysis. FEMA: Fixed-effects meta-analysis.

|  | **Mean** | **Median** | **2.5% HPD** | **97.5% HPD** |
| --- | --- | --- | --- | --- |
| **e^μ^** | 1.01 | 1.01 | 0.92 | 1.10 |
| **𝛽** | 0.03 | 0.03 | -0.04 | 0.11 |
| **γ** | -0.01 | -0.01 | -0.21 | 0.20 |

**S5 Table:** **Posterior statistics for the model coefficients of the REMR with baseline severity as a predictor.**

Mean, median and 95% HPD interval for the *e^μ^*, 𝛽 and γ coefficient of the meta-regression model, where: $VR\approx e^{\mu}*{RR}^{\beta}*{{(e}^{BL})}$ ($lnVR\approx\mu+ \beta*lnRR+\mathbf{*}BL$). VR: Variability ratio, RR: Response ratio, BL: Baseline severity.

*Insert supplementary figure 3a here*

*Insert supplementary figure 3b here*

**S3 Fig: Given a VR of 1.02,** **treatment effect heterogeneity is low, if response and treatment effect are uncorrelated.**

Change score of 1000 simulated patients under placebo (blue) and under active treatment (red) for ρ = 0.0 (no correlation between response under placebo and individual treatment effect), SD_TE_= 1.5 and VR = 1.02. In this particular simulation, the values were: SD_TE_ = 1.48 and VR = 1.04.

*Insert supplementary figure 4 here*

**S4 Fig:** **Treatment effect heterogeneity is low, if response and treatment effect are uncorrelated.**

Individual treatment effect of 100 simulated patients, ρ = 0.0, SD_TE_ = 1.48 and VR = 1.04. Slopes represent individual treatment effect, which has low heterogeneity in this simulation. Blue lines indicated improvement under active treatment, red lines deterioration.

*Insert supplementary figure 5a here*

*Insert supplementary figure 5b here*

**S5 Fig:** **Large treatment effect heterogeneity combined with low VR.**

Change score of 1000 simulated patients under placebo (blue) and under active treatment (red) for ρ = - 0.8, SD_TE_ = 6.5 (6.4 in this simulation) and VR = 0.6. Here, the treatment effect heterogeneity is very large.

*Insert supplementary figure 6 here*

**S6 Fig: Large treatment effect heterogeneity combined with low VR.**

Individual treatment effect of 100 simulated patients, ρ = - 0.8, SD_TE_ = 6.5 and VR = 0.6. Slopes represent individual treatment effect, which has high heterogeneity in this simulation. Blue lines indicate improvement under active treatment, red lines deterioration.
